# Multidrug-resistant *Acinetobacter baumannii*, Russia

**DOI:** 10.3201/eid1304.060755

**Published:** 2007-04

**Authors:** Thierry Naas, Serge Kernbaum, Sophie Allali, Patrice Nordmann

**Affiliations:** * Hôpital de Bicêtre, K.-Bicêtre, France; † American Hospital of Paris, Neuilly-sur-Seine, France

**Keywords:** ESBL, inter-country spread, multidrug resistance, carbapenem resistance, letter

**To the Editor:** During the past decade, nosocomial infections due to multidrug-resistant *Acinetobacter baumannii* have been described with increasing frequency, mostly in intensive care units (ICUs), resulting in therapeutic difficulties (*1*). The main mechanism for resistance to extended-spectrum cephalosporins in *A. baumannii* is attributed to the overexpression of chromosome-encoded cephalosporinases or to plasmid-encoded Ambler class A, B, and D β-lactamases (*2*). *A. baumannii* that produce PER-1 extended-spectrum β-lactamase (ESBL) are rarely isolated outside Turkey and remain susceptible to carbapenems (*3*). Here we describe what we believe is the first ESBL-producing *A. baumannii* isolate resistant to carbapenems and the first characterization of a PER-1 *A. baumannii* isolate from Russia, further supporting the emergence and dissemination of PER-1 *A. baumannii* strains in eastern Europe and outside Turkey (*3**,**4*).

On April 17, 2005, a 79-year-old-man was hospitalized in the cardiology ward of a private hospital in Moscow, Russia, with cardiac arrhythmia and a pulmonary infarction subsequent to a pulmonary embolism. After 1 week, he was transferred to the ICU for multiple organ failure related to a nosocomial infection caused by an *A. baumannii* strain susceptible to several antimicrobial drugs, including imipenem (with positive lung aspiration and blood cultures). He received imipenem and amikacin at high doses. On May 5, 2005, he was transferred to the internal medicine ward of the American Hospital of Paris, Neuilly-sur-Seine, France. On the day of admission, bacterial cultures taken from sputum showed a multidrug-resistant *A. baumannii* MOS-1 strain, susceptible only to colistin and rifampin. The patient received intravenous and aerosolized colistin 3 times a day plus rifampin at 1,200 mg/d so that he could return to Russia. Rapid identification of *A. baumannii* MOS-1, increased awareness as a result of a French national alert signaling the emergence of ESBL VEB-1–producing *A. baumannii* (*5*), and implementation of strict barrier precautions prevented dissemination of this strain. No other multidrug-resistant *A. baumannii* isolate with a similar resistance profile has been isolated in the hospital before, during, or after this period.

*A. baumannii* MOS-1 was susceptible to colistin and rifampin only, and no synergy image could be observed between clavulanic acid and cefepime or ceftazidime discs on a routine antibiogram performed by the disc diffusion method. Only the use of cloxacillin-containing Mueller-Hinton agar plates (200 µg/mL) to inhibit the activity of the naturally occurring cephalosporinase (AmpC) allowed detection of a synergy image, signature of the presence of an ESBL (*5*). MICs for imipenem, determined by the agar dilution method (*6*), were >64 µg/mL. Clavulanic acid addition (2 µg/mL) decreased ticarcillin MIC from >512 to 256 µg/mL and ceftazidime MIC from 512 to 128 µg/mL but did not affect MIC of imipenem.

Genes coding for ESBLs and for class B and D carbapenemases were sought by PCR as previously described (*4**,**5*). Primers used for detection of TEM and PER β-lactamases gave 894-bp and 825-bp PCR products, respectively (*4*). Sequence analysis showed identity with *bla*_TEM-1_ and *bla*_PER-1_ genes (*4*). Results of isoelectric focusing showed 3 isoelectric point values (5.3 for PER-1, 5.4 for TEM-1, and >8.5 for AmpC) in *A. baumannii* MOS-1 (*4*). A crude β-lactamase extract of that isolate had no significant imipenem hydrolysis activity, which suggests that the carbapenem resistance may have emerged through a nonenzymatic mechanism such as mutations in porins (*7*). *Bla*_PER-1_ gene in *A. baumannii* MOS-1 was located on a composite transposon, Tn*1213*, identical to that characterized by Poirel et al. (*8*). Attempts to demonstrate plasmids or transfer genes encoding TEM-1 or PER-1 failed (data not shown), which suggests that the genes were chromosomally encoded. *A. baumannii* MOS-1 was not clonally related to well-characterized PER-1 *A. baumannii* strains from Turkey, France, and Belgium (*4**,**9*) (Figure), further supporting genetic heterogeneity of PER-1 *A. baumannii* isolates, even though the immediate genetic environment of *bla*_PER-1_ gene was similar (*8*).

**Figure:**
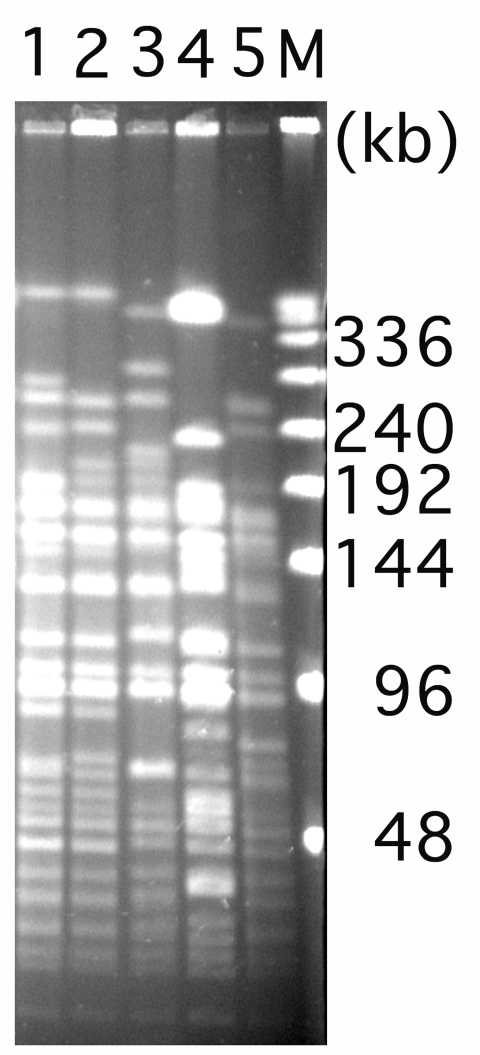
Pulsed-field gel electrophoresis of *Apa*I restricted analysis of *Acinetobacter baumannii* isolates. Lane 1, *A. baumannii* AMA-1 from France (*4*); lanes 2 and 3, *A. baumannii* IST-1 and *A. baumannii* IST-2 from Turkey (*4*); lane 4, *A. baumannii* isolate from Belgium (*4*); and lane 5, *A. baumannii* MOS-1 from Russia (current study). Numbers on the right side of the figure represent the sizes in kb. M, lambda ladder.

The emergence and spread of ESBL-producing *A. baumannii* strains are of concern because they will increase carbapenem use, thus raising the risk for emergence of carbapenem-resistant isolates. *A. baumannii* MOS-1 is, we believe, the first description of an ESBL-producing *A. baumannii* isolate also being resistant to carbapenems. This resistance was likely acquired in vivo under imipenem treatment, but the susceptible strain was not available for strain comparison. This is also the first description of a PER-1 *A. baumannii* isolate from Russia, a country from which little epidemiologic data on antimicrobial drug resistance are available, except for the emergence of ESBLs of CTX-M type in *Enterobactericeae* (*10*).

This study highlights the importance of international patient transfer in the spread of antimicrobial drug resistance, thus emphasizing the need for hospitals to isolate and screen for multidrug-resistant pathogens in all patients admitted to hospitals from foreign countries. This is particularly critical when the foreign country is known for a high prevalence of multidrug-resistant bacteria or when no antimicrobial drug resistance data are available.
